# Highly Hydrophobic Polydimethylsiloxane-Coated Expanded Vermiculite Sorbents for Selective Oil Removal from Water

**DOI:** 10.3390/nano11020367

**Published:** 2021-02-02

**Authors:** Duc Cuong Nguyen, Trung Tuyen Bui, Yeong Beom Cho, Yong Shin Kim

**Affiliations:** 1 Department of Bionano Engineering, Hanyang University, Ansan 426-791, Korea; cuongnguyen@hanyang.ac.kr (D.C.N.); trungtuyen@hanyang.ac.kr (T.T.B.); youngbumcho@naver.com (Y.B.C.); 2 Department of Chemical and Molecular Engineering, Hanyang University, Ansan 426-791, Korea

**Keywords:** expanded vermiculite, PDMS vapor deposition, oil sorbent, oil-collecting

## Abstract

Naturally abundant vermiculite clay was expanded by using an aqueous solution of H_2_O_2_ and its surface was modified with ultra-thin polydimethylsiloxane (PDMS) using facile thermal vapor deposition to prepare an ecologically friendly, low-cost oil sorbent that plays an important role in oil spillage remediation. The resulting PDMS-coated expanded vermiculite (eVMT@PDMS) particles exhibited adequate hydrophobicity and oleophilicity for oil/water separation, with numerous conical slit pores (a size of 0.1–100 μm) providing a great sorption capacity and an efficient capillarity-driven flow pathway for oil collection. Simply with using a physically-packed eVMT@PDMS tube (or pouch), selective oil removals were demonstrated above and beneath the surface of the water. Furthermore, these sorbents were successfully integrated and then applied to the advanced oil-collecting devices such as a barrel-shaped oil skimmer and a self-primed oil pump.

## 1. Introduction

Highly hydrophobic and oleophilic sorbents have attracted extensive interest for oil/water separation tasks. Inspired by the superhydrophobic surfaces of the lotus leaf and the water strider’s leg, various biomimetic materials with superhydrophobicity and superoleophilicity have been studied for high-performance oil sorbents [1,2,3]. The usual strategy for synthesizing such functional materials is preparation of a host matrix with a hierarchical rough surface and subsequent modification of its surface with a low-surface-energy (hydrophobic) moiety. To date, many oil sorbents have been developed with using various host materials, including nanoparticles [4], polymer fabrics [5,6], nanowire membranes [7], metal meshes [8,9], polymer sponges [10,11,12], and carbon-based porous materials [13,14]. Although these sorbents have greatly improved oil separation efficiency and sorption capacity, some crucial issues such as chemical inertness, fire resistance, mechanical stability and ecological friendliness must be resolved before the sorbents are applied to real oil spillage or chemical leakage accidents. Furthermore, since the cleanup processes usually requires a large quantity of sorbents, low-cost mineral [15,16], biomass [17] and waste paper [18] become a promising host matrix of sorbents.

Vermiculite, a clay mineral, can be expanded by thermal or chemical processes to produce a highly porous and lightweight material. The advantages of vermiculite include its chemical inertness, non-toxicity, natural abundancy, and pore-structure controllability, thus providing an opportunity to create ecologically friendly, low-cost sorbents [19,20,21]. Until now, a surface of expanded vermiculite (eVMT) had been commonly modified by impregnation with polymers [22], carnauba wax [23] and porous carbon formed by carbonization of polyglycerol [24] in order to verify the possibility of its use as an oil sorbent. However, these materials exhibited inadequate sorption capacity and oil separation efficiency performance, partly due to the difficulty in control of their porous microstructures.

Commercially available polydimethylsiloxane (PDMS) is widely utilized as a hydrophobic coating agent due to its non-toxicity, adhesiveness, strong hydrophobicity, and reasonable thermal stability. For realizing functional materials for oil/water separation, porous PDMS sponges have been successfully prepared by hard template methods [25,26]. In addition, PDMS-coated metal oxide nanoparticles such as TiO_2_ and ZnO have been combined with metal mesh [27], polymer sponge [28], or cotton fabric [29,30] via dip coating. These nanocomposites are proved as outstanding materials in oil/water separation both in air and under water, which demonstrates the great potential of the PDMS surface modification in preparation of highly hydrophobic and oleophilic sorbents. According to previous studies [4,31,32,33], thermal PDMS vapor deposition is known to result in the formation of a conformal and ultrathin PDMS layer with a thickness of 3–5 nm on various substrates, but this is rarely achieved by conventional wet-chemical processes.

Recently, novel oil-collecting devices based on oil-absorbing and water-repelling sorbents have been proposed to minimize the usage amount of sorbent and reduce the disposal of oil-soaked waste. One example is a barrel-shaped oil skimmer with a superhydrophobic-superoleophilic metal meshed cap [34,35]. The inlet cap is partly immersed in the oil spill and put in contact with both floating oil and underneath water. The oil-permeable mesh selectively collects hydrophobic organic chemicals into a container. This device can greatly simplify the oil collection process and decrease the amount of oil sorbents required because the sorption capacity relies on the volume of the container rather than amount of sorbent. Another innovative oil-collecting device is an oil pump that selectively pumps the oil absorbed by sorbents away from an oil-spill site [11,12]. This self-primed pumping strategy can greatly shorten the process time required for oil cleanup and recovery. Hydrophobic nanomaterial-modified polymer sponges [11,12,36] and biomass-derived carbon aerogels [37] had been successfully utilized as a sorbent of the self-primed oil pump.

In this work, vermiculite was delicately exfoliated by using an aqueous solution of H_2_O_2_ to produce porous, capillary-dense and lightweight support materials for oil sorbents. Through the surface modification with ultra-thin, low-surface-energy PDMS by facile thermal vapor deposition, hydrophilic eVMT was transformed into a hydrophobic and oleophilic sorbent capable of selectively absorbing oils from water, preserving its complex capillary structures. A column packed with PDMS-coated expanded vermiculite particles (eVMT@PDMS) was found to exhibit remarkable water repellency and good oil permeability. Due to the absorbing selectivity, integrated eVMT@PDMS forms (a physically-packed tube and pouch) were successfully applied to both a barrel-shaped oil skimmer and a self-primed oil pump. To the best of our knowledge, the particulate-based integrated sorbent has been first applied to the oil-collecting devices instead of the monolithic sorbents based on metal meshes or polymer sponges. The clay-based oil sorbent can provide a superior flexibility and a competitive price in realizing practical oil-collecting systems for large-scale oil spills and chemical leaks.

## 2. Materials and Methods

### 2.1. Chemicals

Crude vermiculite of micro-grade (Palabora mining company, Phalaborwa, South Africa) was supplied by Shinsung Mineral (Seongnam, Korea). Ethanol, *n*-hexane, and 30.0 wt.% H_2_O_2_(aq) were purchased from Daejung Chemicals & Metals (Siheung, Korea). Oil Red O, diiodomethane and chloroform were acquired from Sigma-Aldrich. PDMS prepolymer (Sylgard 184A, Dow Corning, Midland, MI, USA) and a blue food dye solution (Food Colors, Kroger, Cincinnati, OH, USA) were purchased from a local agent and stationary store, respectively. All chemicals of reagent grade were used without any further purification.

### 2.2. Preparation and Characterization of PDMS-Coated Expanded Vermiculite

Crude vermiculite was classified according to its planar size and thickness by using a sieving machine and a weight-based separation process, respectively. The vermiculite sample with an average planar size of 0.50 ± 0.15 mm and a thickness of 0.11 ± 0.10 mm was used in this work and chemically treated to prepare an expanded form. Typically, 10 g of vermiculite was spread in a glass Petri dish containing 60 mL of H_2_O_2_ aqueous solution. The glass dish was covered with a lid and then placed in an oven at 60 °C for 1 h. After taking out the sample from the solution, it was treated in a microwave oven (KR-S341T, 1 kW, Daewoo, Gwangju, Korea) for 10 min in order to quickly evaporate the remaining liquid. The fast microwave drying allowed production of highly expanded vermiculite via accompanying secondary expansion. The sample was further dried in a vacuum oven at 150 °C for 1 h, and stored in a vial for next experiments. For the formation of a very thin PDMS layer, 3.0 g of eVMT was placed inside a reaction chamber with a small vessel containing 2.0 g of the PDMS prepolymer. The reactor was covered with a lid and kept in an oven at 230 °C. PDMS-coated vermiculite samples were typically prepared with a reaction time of 8 h.

Surface morphology of samples was observed by using scanning electron microscopy (SEM, S-4800, Hitachi, Tokyo, Japan) under an electron beam with an accelerating voltage of 15 or 20 keV after the deposition of a platinum nano-film. Fourier transform infrared (FTIR) spectra were obtained by a Spectrum Two spectrometer (Perkin-Elmer, Waltham, MA, USA) with an attenuated total reflectance sampling accessory (Miracle ATR, Pike, Madison, WI, USA) in the range of 500–4000 cm^−1^. Nitrogen sorption (Belsorp-mini II, MicrotracBEL, Osaka, Japan) and mercury porosimetry (AutoPore IV 9500, Micromeritics, Norcross, GA, USA) were used to characterize pore size distribution and specific surface areas. A static water contact angle (WCA) was measured on vermiculite particles attached on an adhesive tape using a goniometer (CAM200, KSV Instruments, Helsinki, Finland). The contact angle for diiodomethane was also measured to evaluate its surface free energy. The contact angle measurement was performed three times at different positions to get an average value.

### 2.3. Evaluation of Liquid Uptake Behaviors

Liquid uptake behaviors were investigated by a capillary penetration method. 1.0 g of eVMT@PDMS was added into a glass tube (D = 8 mm, L = 300 mm), and the vermiculite-containing tube was dropped approximately 300 times from a height of 200 mm in order to pack the sample. The resulting packed column was connected to a scaffold, which was placed on a balance (GX-200, A&D, Tokyo, Japan). A beaker with test liquid was slowly moved up until the bottom of the column was immersed to approximately 10 mm. The uptake mass induced by liquid absorption into the packed tube was recorded automatically by a computer. These experiments were repeated 6 times to obtain an averaged time-dependent uptake mass profile. In addition, sorption capacity was also determined according to a previously reported method for particle samples [38]. Typically, a stainless-steel mesh cell was charged by a 1.0 g eVMT@PDMS sample, fully immersed in test liquid for 5 min and shaken at 60 rpm. The cell was lifted up and excess liquid drained off. Shortly afterward, the weight of absorbed sample (*M_abs_*) was measured by a balance, while the dry mass (*M_dry_*) was quantified after drying in a vacuum oven at 150 °C for 1 h. The sorption capacity, defined by a relative weight of absorbed liquid to sorbent, was calculated by the Equation (1). Three measurements were performed to determine the average sorption capacity:

*Sorption capacity* = (*M_abs_* − *M_dry_*)/*M_dry_*. (1)

### 2.4. Selective Oil Removal Tests 

Three different types of oil removal tests were performed to evaluate the selective oil separation capability of eVMT@PDMS sorbent from water. First, an oil-absorbing column was fabricated by packing 1.8 g of eVMT@PDMS inside a plastic tube (D = 19 mm, L = 60 mm). Using the sorbent tube, floating *n*-hexane and underwater chloroform were selectively removed from water. Next, a barrel-shaped oil-collecting skimmer was fabricated by using a circular eVMT@PDMS pouch (Φ = 47 mm) as a flow inlet. A skin of the pouch consisted of nylon mesh with 200 µm rectangular openings, and a 50 mL glassware was used as a container. This cylinder-like device spontaneously absorbed 20 mL of *n*-hexane from the surface of the water, and then collected it into the container. Finally, a vacuum aspirator was connected to the side glass pipe (D = 6 mm) of a T-shaped eVMT@PDMS-packing tube for continuous suction of oil from the water surface (see Figure S1 in the Supplementary Material). The pumping pipe was positioned at the middle point of the sorbent tube, and the pipe tip was fixed at the cross-sectional center of the tube.

## 3. Results and Discussion

### 3.1. Material Properties of eVMT@PDMS

**Figure 1:**
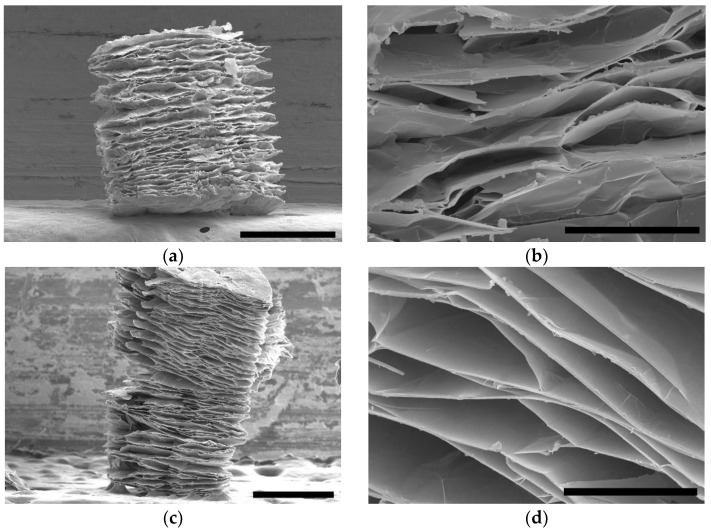
SEM images of (**a**,**b**) eVMT and (**c**,**d**) eVMT@PDMS samples. The black scale bars in (**a**) and (**c**) correspond to 500 µm while the scale bars in (**b**) and (**d**) are 50 µm.

Microstructures of the initially prepared and the PDMS-coated eVMT samples were inspected by SEM. The surface morphology of the eVMT is shown in Figure 1a. The magnified image shown in Figure 1b clearly exhibits a partially exfoliated concertina-like shape with various conical slit gaps having an opening size of 1–100 µm. The expansion ratio, defined as a relative volume ratio of eVMT to non-expanded crude vermiculite, was evaluated to be 11 from the specific gravity measurements. This value is approximately four times higher than that of vermiculite thermally expanded at 1000 °C [38], indicating that the combination of 60 °C H_2_O_2_ treatment and subsequent microwave heating results in extensive exfoliation of vermiculite. After being exposed to thermal PDMS vapor at 230 °C for 8 h, the expanded structure was still evident, as shown in Figure 1c,d. The delaminated surface seems to be slightly smoother after PDMS treatment. However, no perceivable structural change was observed, except for the surface roughness.

**Figure 2:**
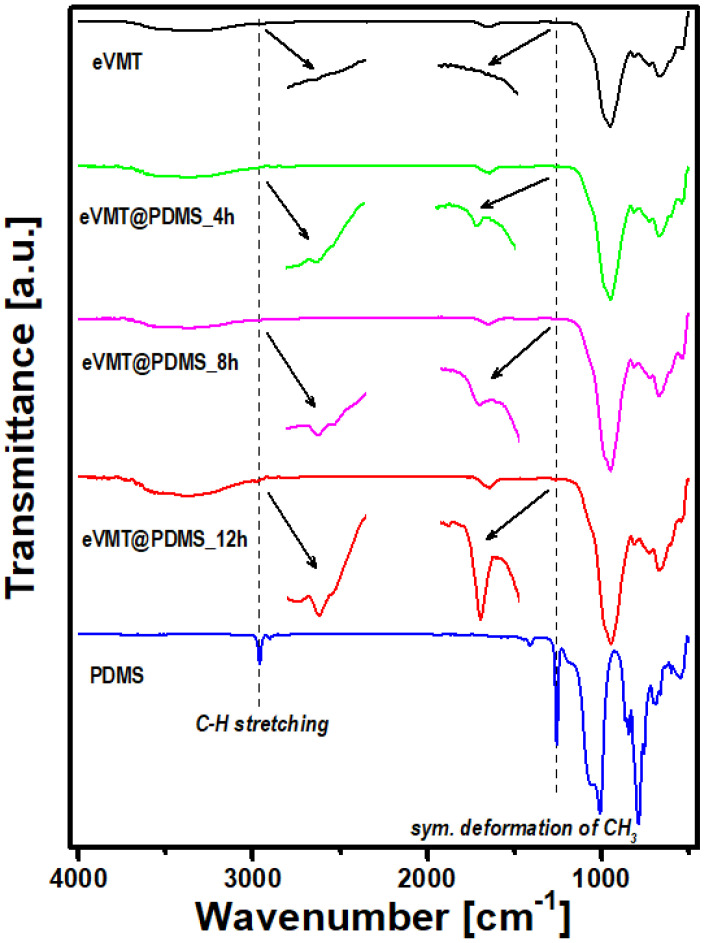
FTIR spectra of eVMT, PDMS, and three eVMT@PDMS samples prepared with the three different deposition times of 4, 8 and 12 h.

The PDMS-treated samples were further analyzed by FTIR to confirm the existence of organic functional groups. Figure 2 shows FTIR spectra of eVMT and three eVMT@PDMS samples prepared with different deposition times (4, 8, and 12 h) in addition to that of PDMS. The expanded vermiculite exhibited characteristic bands originating from the aluminosilicate structure in the low-energy region of 1200–500 cm^−1^: Si-O stretching at 950 cm^−1^, Al-O-H bending at 814 cm^−1^, and deformation vibrations of Al-O-Si at 730, 664, and 530 cm^−1^ [39,40]. In the eVMT@PDMS spectra, two additional weak peaks appeared at 2981 and 1264 cm^−1^ together with the characteristic vermiculite peaks (see the magnified spectra), and their intensities were enhanced as deposition time increases. These new peaks are also evident in the PDMS spectrum, being assigned to the stretching of C-H bonds and the symmetric deformation of -CH_3_ in PDMS, respectively [4,31]. These observations indicate that PDMS was successfully incorporated into expanded vermiculite during the thermal vapor deposition, and that the amount of PDMS could be controlled by the deposition time.

**Figure 3:**
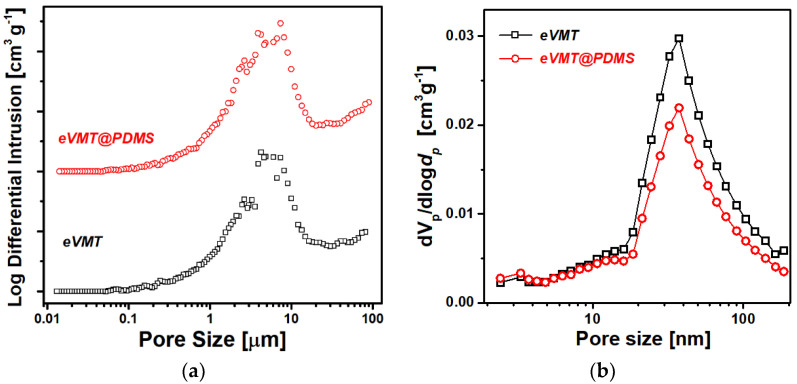
Pore size distributions of eVMT and eVMT@PDMS obtained from (**a**) Hg porosimetry and (**b**) N_2_ adsorption isotherm.

Both Hg porosimetry and N_2_ sorption measurements were carried out to characterize pore size distributions of the eVMT and eVMT@PDMS samples in a wide range of sizes from 2 nm to 100 µm. The pore size distributions probed by the Hg porosimeter are shown in Figure 3a. The samples exhibit a broad pore size distribution in the range of 0.1–100 µm with one peak at around 5 µm. These pore dimensions well match with those of the conical slit gaps observed by SEM (Figure 1a,b). There was no discernible difference in pore size distribution between the two samples, which implies that the capillary structure larger than 0.1 µm was negligibly altered by PDMS coating.

N_2_ sorption curves of the eVMT and eVMT@PDMS samples exhibited type IV isotherms with one hysteresis loop, which can be attributed to capillary condensation in the mesopores. Figure 3b shows pore size distributions of the two samples in the range of 2–200 nm, calculated by Barrett-Joyner-Halenda (BJH) analysis. After PDMS coating, the differential volume of pores larger than approximately 10 nm decreased moderately. The decrease was well consistent with the reductions in specific surface area, average pore diameter, and total pore volume obtained from Brunauer-Emmett-Teller (BET) analysis (see Table S1). A mean thickness of the PDMS-modified layer was estimated to be 2.6 nm from the decrease in average pore diameter, which was in agreement with the previously obtained results of producing PDMS nano-film with a thickness of less than 5 nm on various solid surfaces [31,32,33]. Consequently, the pore size distributions support that the thermal vapor deposition produces an ultra-thin PDMS layer on a vermiculite surface, preserving the complex capillary structures of pristine eVMT.

### 3.2. Wettability and Liquid Uptake Behavior

**Figure 4:**
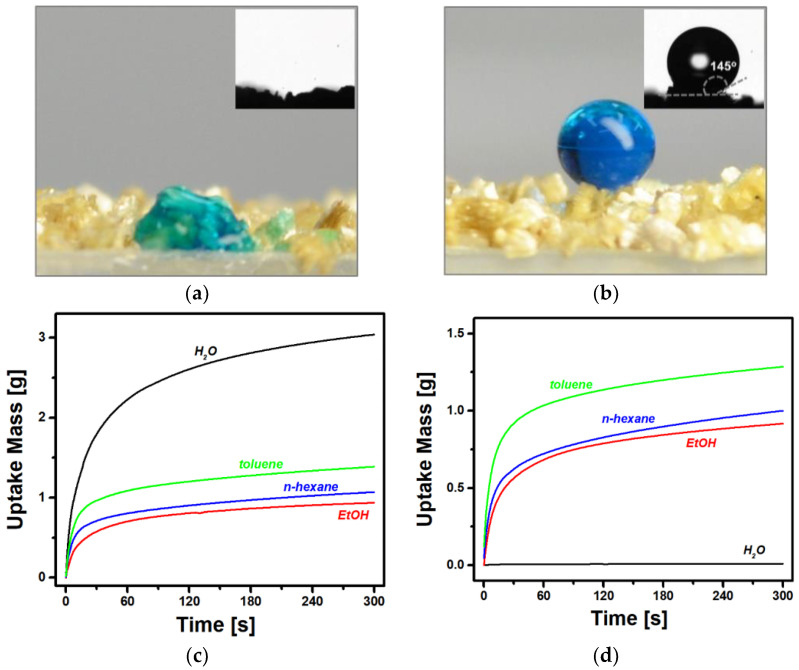
Optical images of (**a**) eVMT and (**b**) eVMT@PDMS after dispensing a 2 µL water droplet on the surface. The insets display water contact angles and water was stained by a blue dye. Time-dependent uptake mass variations for four different liquids along the capillary tubes packed with (**c**) eVMT and (**d**) eVMT@PDMS particles.

The change in wetting property after PDMS deposition was examined on a surface of vermiculite particles positioned on an adhesive tape using the sessile drop method. Figure 4a,b show optical images of water droplets on the surfaces of eVMT and eVMT@PDMS, respectively. The bare sample quickly absorbed the water droplet in less than 1 s, resulting in WCA = 0°. This observation is in good agreement with previously reported superhydrophilicity of vermiculite [41]. Meanwhile, the PDMS-coated sample exhibited hydrophobic characteristic of WCA = 145 ± 8°. A contact angle is generally determined by both surface roughness and interfacial energies among solid, liquid, and gas phases. Because there was no change in surface morphology after PDMS coating, hydrophobicity is mainly regarded as a consequence of the interfacial energy change induced by the surface modification of PDMS with a low surface energy. The interpretation is in good agreement with the observation that surface free energy decreases from 81.4 to 16.6 mN/m after the coating, evaluated by the simple Owens-Wendt method [42]. The obtained WCA of eVMT@PDMS was greater than the previously reported values (WCA = 99 − 138°) on the surface of PDMS substrates [43]. The WCA enhancement suggests the involvement of the slit-shaped structure of expanded vermiculite resulting in highly rough surface morphology. On the other hand, both the eVMT and eVMT@PDMS layers were found to well absorb a *n*-hexane droplet in additional experiments, which indicates that eVMT was endowed with hydrophobicity by the PDMS surface modification.

Liquid penetration rates were measured to characterize the wetting properties of porous particles using a cylindrical glass tube packed with vermiculite samples, more quantitatively. Figure 4c,d show variation curves of the uptake mass of liquid as a function of time for the eVMT and eVMT@PDMS samples, respectively. The water uptake mass of eVMT increased rapidly in the early stage of wicking and then exhibited a gradual slowdown in penetration rate, but there is no water wicking in eVMT@PDMS. The PDMS deposition resulted in switching the water-wetting property from permeability to repellency. The water-repellent property persisted even when the packed tube was immersed to a depth of 15 cm, corresponding to an intrusion pressure of 1.47 kPa. On the other hand, the uptake mass profiles of the organic liquids (ethanol, *n*-hexane, and toluene) were similar irrespective of PDMS coating. A dynamic contact angle (*θ*) of the sample in an early wicking period was evaluated from an uptake mass profile using a modified Washburn equation [44,45]:

(2) $$m^{2}\;=\frac{\;\;C\rho^{2}\;\gamma \;\cos\;\theta \;\;\;}{\;\eta }\;t,$$

where *m* is the mass of an imbibed liquid, *C* is the geometric factor, *ρ* is the density of liquid, *γ* is the surface tension, *η* is the viscosity of liquid, and *t* is the penetration time. The time-dependent variations of squared uptake mass during an initial liquid wicking period of 0–15 s are shown in Supplementary Material Figure S2. The *C⋅*cos *θ* value can be determined from the slope of a linear plot of *t* vs. *m^2^* for a test liquid with known values of *ρ*, *γ* and *η*. To calculate a dynamic contact angle from *C⋅*cos *θ*, it is necessary to establish a complete wetting (*θ = 0°*) reference system. The water wetting of eVMT was used as the reference because its *C⋅*cos *θ* value was the largest and its WCA was zero in the sessile drop measurement, as mentioned above. The same geometric factor *C* was assumed to calculate the dynamic contact angles. Results obtained using eVMT and eVMT@PDMS columns are summarized in Table 1. The eVMT@PDMS with a wicking slope of zero corresponds to a maximum dynamic contact angle value of 90° in the capillary penetration method, indicating hydrophobic wettability. The PDMS coating resulted in a dramatic change in dynamic water wettability while preserving capillary pore structures. However, the contact angles for ethanol, *n*-hexane, and toluene did not change significantly after PDMS modification. Both eVMT and eVMT@PDMS samples exhibited oleophilicity for all the organic liquids. As a result, the physically packed eVMT@PDMS column demonstrated selective repellency to water against organic chemicals, which is an important requirement for oil-water separation applications.

**Figure 5:**
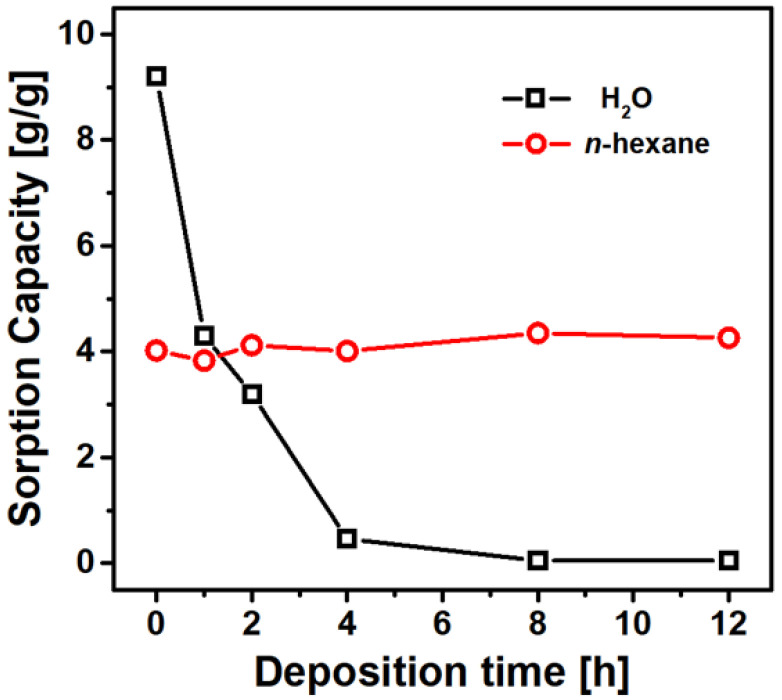
Variations of sorption capacity as a function of PDMS deposition time for water and *n*-hexane.

Furthermore, the sorption capacities of water and *n*-hexane were measured as a function of deposition time to clarify the effect of PDMS content on surface wettability. The water sorption capacity rapidly decayed with increasing deposition time, whereas the *n*-hexane sorption capacity retained a value of around approximately 4.1 g/g (see Figure 5), as expected from the capillary penetration experiments. The water sorption capacity of eVMT@PDMS treated for 4 h was 20 times smaller when compared to that of the non-coated sample. The water sorption became negligible with the samples treated for 8 and 12 h, indicating that the 8 h PDMS deposition was enough to prepare a water-repellent sample. The 8 h eVMT@PDMS sample was used as a sorbent in following oil removal tests.

### 3.3. Selective Oil Removal from Water

**Figure 6:**
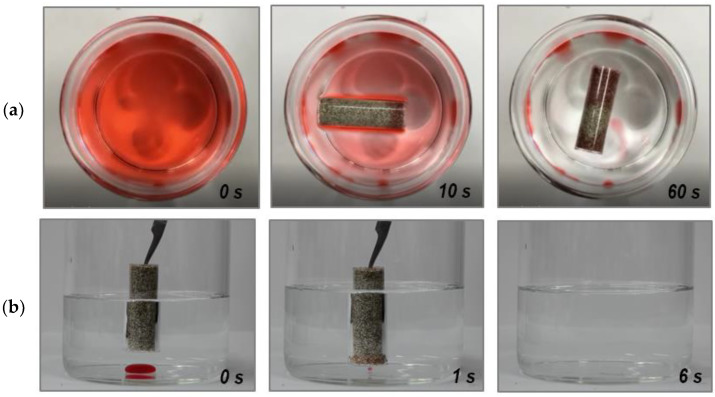
Snapshots taken at different times demonstrating the selective removal of (**a**) *n*-hexane on the surface of water and (**b**) chloroform underwater using the selectively oil-absorbing eVMT@PDMS column. The oils in (**a**) and (**b**) were stained with Oil Red O.

A plastic column packed by eVMT@PDMS sorbents was employed to evaluate the performance of selective oil (hydrocarbon compound) separation from water. Each of Figure 6a,b show three snapshots taken at different times, displaying selective removal of floating *n*-hexane and underwater chloroform, respectively. Full moving pictures are provided as Movies S1 and S2 in the Supplementary Material. In the case of *n*-hexane removal, the lightweight column floated in the water as would a ship. The red-stained *n*-hexane gradually pervaded the eVMT@PDMS-packing tube through the permeable top and bottom sides of the column. Most of the oil was spontaneously absorbed by the capillary action in 60 s, which disclosed the selective oil-absorbing property of eVMT@PDMS against water. A red-colored column area (oil-soaked region) became constant after the complete oil absorption, and its fraction was approximately 70%. When considering the used weights of *n*-hexane (5 g) and sorbent (1.8 g), the oil removal capacity of the sorbent tube was in agreement with the value expected from the obtained sorption capacity of 4.1 g/g. When the sorbent tube is used, its oil-absorbing volume is limited by the sorption capacity of sorbent. The removal time can be greatly reduced if the sorbent tube is in motion and its side wall is also permeable. On the other hand, the same tube was vertically submerged in water to remove small amounts of chloroform at the bottom of a beaker. When the underwater chloroform droplet was contacted with the bottom side of the tube, it was rapidly absorbed into the tube in less than 1 s. This removal process is similar to picking up small objects with tweezers. In additional test experiments, the packed column could be replaced with an eVMT@PDMS pouch fully enclosed by permeable nylon mesh. These results demonstrate that the physically packed eVMT@PDMS column or pouch can be utilized as an integrated sorbent for selective oil removal from water.

**Figure 7:**
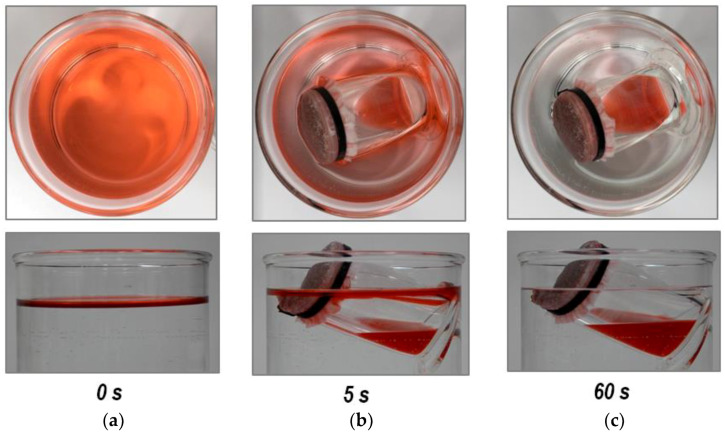
Snapshots of the barrel-shaped oil skimmer taken at different times of (**a**) 0 s, (**b**) 5 s, and (**c**) 60 s. They display the operation process of collecting *n*-hexane (dyed red) from the surface of water. The upper and bottom images correspond to top and side views, respectively.

The eVMT@PDMS pouch was applied to an inlet of a barrel-shaped oil skimmer. The pictures shown in Figure 7 display the oil-collecting process of the oil skimmer: *n*-hexane (dyed red) present above water is selectively absorbed by the eVMT@PDMS inlet and spontaneously transported to the inside of a glass bottle (see Movie S3 in the Supplementary Material). There was no water transportation into the bottle, indicating adequate water repellency of the eVMT@PDMS pouch. A bottle with a massive pedestal was used to ensure the bottle base was lower than the inlet. The slightly tilted, floating placement of the barrel-shaped skimmer is required for its normal operation because the inlet must make contact with the floating oil. The tilted positioning is necessary for the gravity-driven drain from the oil-soaked sorbent [34,35]. In this test, the oil skimmer fabricated with 1 g of eVMT@PDMS removed and gathered 20 mL of *n*-hexane for 60 s into the bottle. The removal capacity corresponds to 13.1 g/g that is greatly higher than that of the sorbent tube. Since the capacity is mainly limited by the inner volume of a container, it can be enhanced with using a larger barrel. This result implies that inexpensive mineral-based particulate sorbents can be utilized to fabricate a smart oil-removal skimmer.

**Figure 8:**
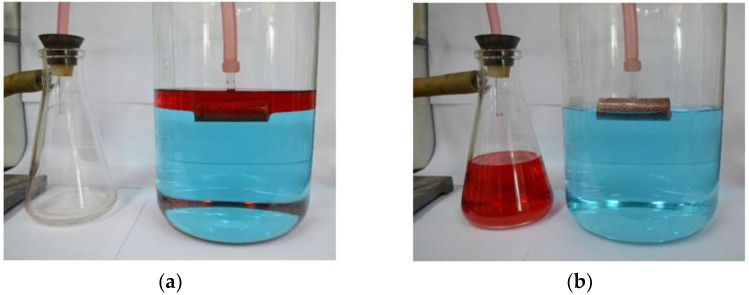
Optical pictures of the self-primed oil pump taken (**a**) before and (**a**) after its operation. The 385 mL of *n*-hexane (dyed red) on the surface of water (dyed blue) was selectively transferred to the collecting flask by a vacuum aspirator during an operation time of 120 s.

Continuous oil suction from oil-water systems was performed by using a T-shaped eVMT@PDMS tube connected to a vacuum aspirator. Figure 8a,b depict the oil pump at an initial state and after oil separation, respectively. Its continuous operation is shown in Movie S4 in the Supplementary Material. When the aspirator was turned on, the red-colored *n*-hexane on the surface of the water (dyed blue) started to move to the collecting flask along the upward pumping pipe without carrying water. Both end faces of the eVMT@PDMS packing tube acted as a flow gate, and the hydrophobic-oleophilic column showed selective permeability to oils and air against water. The oil level decreased gradually during the pump operation. When the entrance gate was partially exposed to the atmosphere, the oil and air flowed together into the collecting flask to a state of complete oil removal. It took approximately 120 s to remove 385 mL of *n*-hexane from the surface of the water and collect it within the container, corresponding to an oil-removal rate of 3.2 mL/s. Theoretically, the sorption capacity of the self-primed oil pump is infinite, because the removal amount is proportional to pumping time rather than amount of sorbent. The oil removal rate can be regulated and increased with a sophisticated oil pump. Consequently, this simple demonstration reveals the potential for the physically packed eVMT@PDMS tube in an active oil-pumping device to remedy for large-scale oil spillages.

## 4. Conclusions

Chemically expanded vermiculite clay minerals were surface-modified with nanometer-thick PDMS layers by using simple thermal vapor deposition, producing a highly hydrophobic-oleophilic sorbent. The particulate eVMT@PDMS containing various conically slitted capillary pores exhibited a capability for highly efficient oil separation of oil/water mixtures by means of spontaneous and rapid imbibition of oil. This inexpensive clay-based material has the potential to become a practical oil sorbent due to its thermal stability, chemical inertness, and low environmental impact. In addition, its integrated sorbent forms (a physically-packed eVMT@PDMS tube and pouch) were fabricated and successfully utilized as a selectively oil-filtering component in both a barrel-shaped oil skimmer and a self-primed oil pump, instead of the previously used monolithic sorbents such as metal meshes and polymer sponges. Because these advanced oil-collecting devices greatly simplify the oil collection process and shorten cleanup time, the new sorbent forms based on inexpensive eVMT@PDMS particles present a flexibility to develop a smart oil-collecting device or system appropriate for large-scale oil spills and chemical leaks.

## Figures and Tables

**Table 1:** Summarized results obtained from the capillary penetration experiments.

Sample ID	Wetting Liquid	Wicking Slope ^1^ (g^2^/s)	*C*⋅cos *θ* (× 10^−16^ m^5^)	Dynamic Contact Angle, *θ* (°)
eVMT	water	0.1210	17.0	0.0 ^2^
	ethanol	0.0146	12.0	45.2
	*n*-hexane	0.0248	9.4	56.4
	toluene	0.0461	12.3	43.8
eVMT@PDMS	water	0.0000	0.00	90.0
	ethanol	0.0140	11.5	47.6
	*n*-hexane	0.0188	7.1	65.3
	toluene	0.0401	10.7	51.1

^1^ Obtained from the linear plot between squared uptake mass and a time. ^2^ Water was assumed to be *θ* = 0 on the eVMT surface.

## Data Availability

The data presented in this study are available on request from the corresponding authors.

## Supplementary Materials

The following are available online at https://www.mdpi.com/2079-4991/11/2/367/s1, Figure S1: A picture showing an experimental setup to pump *n*-hexane on the surface of water through the eVMT@PDMS-packing tube, Figure S2: Time-dependent variations of squared uptake mass for four different liquids along the capillary tubes packed by (a) eVMT and (b) eVMT@PDMS particles, Table S1: Specific surface area, average pore diameter, and total pore volume obtained from BET analysis, Video S1: Selective removal of *n*-hexane on the surface of water using the eVMT@PDMS column, Video S2: Selective removal of chloroform underwater using the eVMT@PDMS column, Video S3: A side-view video of a barrel-shaped oil skimmer, collecting *n*-hexane (dyed red) from the surface of water in a speed of 2×, Video S4: Transfer of *n*-hexane (dyed red) on the surface of water (dyed blue) to a collecting flask by an aspirator in a speed of 4×.
