# Supplementary material for: Highly Hydrophobic Polydimethylsiloxane-Coated Expanded Vermiculite Sorbents for Selective Oil Removal from Water
Source: Nanomaterials (Basel). 2021 Feb 2;11(2):367. doi: 10.3390/nano11020367 (PMC7912906; doi:10.3390/nano11020367)
Supplement: Supplementary file 1 [file nanomaterials-11-00367-s001.zip › Supplementary v0_20210111 (NM).docx]

**Supplementary Materials**

**Highly hydrophobic polydimethylsiloxane-coated expanded vermiculite sorbents for selective oil removal from water**

**Duc Cuong Nguyen^1^, Trung Tuyen Bui^1^, Yeong Beom Cho^1^, and Yong Shin Kim^2,^**^†^

^1^ Department of Bionano Engineering, Hanyang University, Ansan 426-791, Republic of Korea

^2^ Department of Chemical and Molecular Engineering, Hanyang University, Ansan 426-791, Republic of Korea

^†^ Authors to whom correspondence should be addressed.

E-mail: [yongshin@hanyang.ac.kr](mailto:yongshin@etri.re.kr); Tel.: +82-31-400-5507; Fax: +82-31-400-5457

**Table**

**Table S1.** Specific surface area, average pore diameter, and total pore volume obtained from BET analysis.

| Sample ID | Specific surface area [m^2^/g] | Average pore diameter [nm] | Total pore volume [cm^3^/g] |
| --- | --- | --- | --- |
| eVMT | 4.76 | 17.0 | 0.020 |
| eVMT@PDMS | 4.26 | 14.4 | 0.015 |

**Movies**

**Movie S1**. Selective removal of *n*-hexane on the surface of water using the eVMT@PDMS column.

**Movie S2.** Selective removal of chloroform underwater using the eVMT@PDMS column.

**Movie S3.** A side-view video of a barrel-shaped oil skimmer, collecting *n*-hexane (dyed red) from the surface of water in a speed of 2X.

**Movie S4.** Transfer of *n*-hexane (dyed red) on the surface of water (dyed blue) to a collecting flask by an aspirator in a speed of 4X.

**Figures**

**
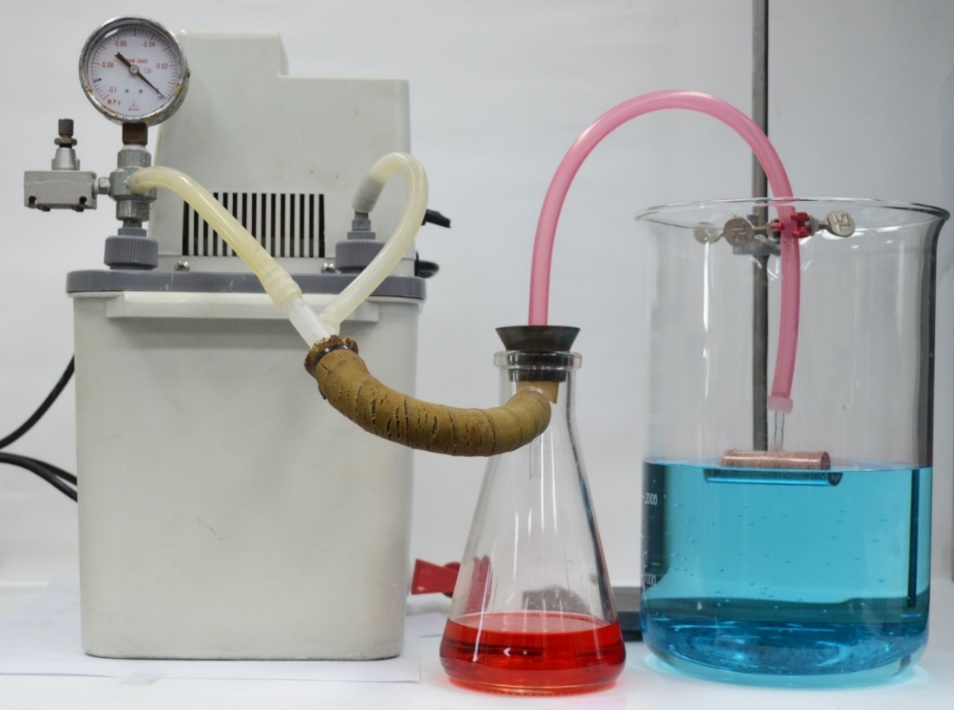
**

**Fig. S1.** A picture showing an experimental setup to pump *n*-hexane on the surface of water through the eVMT@PDMS-packing tube.

1. **(b)**


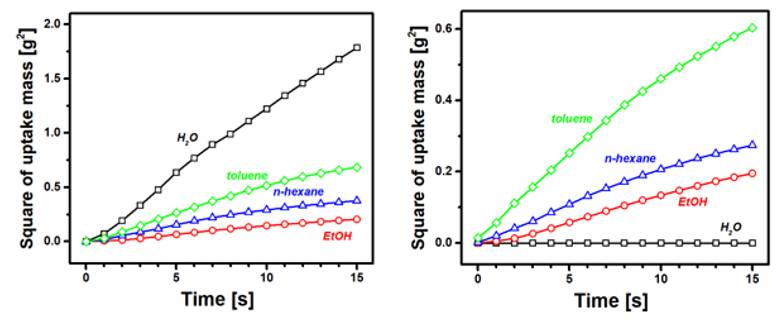
 **Fig. S2.** Time-dependent variations of squared uptake mass for four different liquids along the capillary tubes packed by (a) eVMT and (b) eVMT@PDMS particles.
